# Celastrol-Loaded Hyaluronic Acid/Cancer Cell Membrane Lipid Nanoparticles for Targeted Hepatocellular Carcinoma Prevention

**DOI:** 10.3390/cells13211819

**Published:** 2024-11-04

**Authors:** Peng He, Manshu Zou, Chanjuan Zhang, Yaning Shi, Li Qin

**Affiliations:** 1 Laboratory of Stem Cell Regulation and Application of Traditional Chinese Medicine, School of Pharmacy, Hunan University of Chinese Medicine, Changsha 410208, China; 20223702@stu.hnucm.edu.cn (P.H.); zcj@hnucm.edu.cn (C.Z.); syn@hnucm.edu.cn (Y.S.); 2 Science and Technology Innovation Center, Hunan University of Chinese Medicine, Changsha 410208, China; zoumanshu@hnucm.edu.cn

**Keywords:** homotypic cell membrane, apoptosis, celastrol, liposome, hepatocellular carcinoma

## Abstract

Hepatocellular carcinoma (HCC) is the third leading cause of cancer-related deaths worldwide, and its prevention and treatment face severe challenges. It is crucial to improve the targeting of drugs on tumor cells and tissues. Celastrol (CeT), as an active ingredient of traditional Chinese medicine, possesses strong antitumor effects, especially in triggering apoptosis of HCC. However, due to its toxicity and lack of targeting, its application is greatly limited. HMCLPs, a nano-biomimetic platform carrying CeT with controllable drug release, enhanced targeting, and immunocompatibility, were developed for the first time, which can be used for the treatment of HCC. By utilizing homologous cell membranes and hyaluronic acid (HA), HMCLPs can precisely target tumor regions and release CeT in a controlled manner. Both in vitro and in vivo studies have demonstrated that HMCLPs loaded with CeT significantly increased the accumulation of reactive oxygen species (ROS), induced mitochondrial damage, and triggered apoptosis of HCC cells, resulting in effective treatment with minimal adverse reaction. The development of HMCLPs as a nanocarrier system for CeT delivery offers a promising therapeutic strategy for HCC. This innovative approach improves the targeted delivery and bioavailability of CeT, dramatically induces apoptosis in HCC cells, and exerts its powerful antitumor effects while minimizing systemic toxicity. The present study highlights the potential of combining innovative nanocarriers with powerful natural compounds such as CeT to enhance efficacy and reduce toxicity.

## 1. Introduction

Hepatocellular carcinoma (HCC) accounts for 90% of primary liver cancers and is the third leading cause of cancer-related death worldwide, with a five-year survival rate of only 18% [1]. HCC can be treated through surgical resection, liver transplantation, hepatic arterial infusion therapy, systemic chemotherapy, and so on. Despite these options, most HCC patients still require long-term chemotherapy, and sorafenib is typically applied for advanced HCC patients [2,3]. Therefore, drug therapy for HCC faces enormous challenges. Our previous studies have shown that celastrol (CeT), a pentacyclic triterpenoid compound derived from the roots of the traditional Chinese medicine, *Tripterygium wilfordii*, exhibits potent anti-cancer effects on various tumors [4]. Unfortunately, its application is severely limited due to poor solubility, narrow therapeutic window, and potential toxicity [5,6].

Nanodrug Co-Delivery Systems (NDCDS) are advanced nanotechnology-based platforms aimed at delivering two or more therapeutic agents simultaneously to a specific target, such as cancer cells or other diseased tissues. These systems use nanoscale carriers like liposomes, polymeric nanoparticles, micelles, dendrimers, or solid lipid nanoparticles to encapsulate multiple drugs. Encapsulation of these drugs can reduce adverse reactions and improve therapeutic efficacy by decreasing toxicity and enhancing targeting specificity [7,8]. Recently, various nanoscale carriers with excellent tumor targeting capabilities, biosafety, and enhanced anti-cancer activity have been developed for drug delivery, among which liposomes are the most common. These drug carriers are non-toxic and biodegradable, with low immunogenicity and good biocompatibility [9,10]. Liposomes can encapsulate hydrophobic or hydrophilic drugs. However, ordinary lipid nanoparticles lack targeting specificity. Therefore, it is critical to prepare targeted nanoparticles [11,12]. Currently, there are two main targeting strategies: passive targeting based on enhanced permeability and retention (EPR) effect and active targeting through ligand modification [13]. Our subsequent research focused on using liposomes and CeT to optimize these targeting approaches.

HCC cells usually overexpress the cluster of differentiation CD44, a cell adhesion molecule with a molecular weight of 82 kDa. CD44 is a receptor that plays a crucial role in tumor cell proliferation, invasion, and lymph node metastasis [14,15]. Notably, CD44 can specifically bind to hyaluronic acid (HA), a polymer known for its excellent biocompatibility, biodegradability, and lack of immunogenicity. HA exhibits a high affinity for CD44 overexpressed on tumor cells. When HA is conjugated to drugs, it can promote receptor-mediated endocytosis, actively target lesion sites, reduce toxicity to normal cells, and improve the therapeutic index [16]. Due to its properties, HA is commonly used in biomimetic cell membrane-camouflaged drug delivery systems, which have garnered widespread attention in biomedical applications for their enhanced biocompatibility, low immunogenicity, and active targeting capabilities. Various cell membrane-camouflaged nanoparticles, such as those derived from red blood cells, cancer cells, white blood cells, stem cells, and platelets, have been extensively studied. Cancer cell membranes, in particular, are noteworthy for retaining their original membrane proteins and providing nanoparticles with tumor-targeting capabilities [17,18,19,20,21]. The targeting ability of lipid nanoparticles can be enhanced by modification of HA and cancer cell membranes.

**Scheme 1:**
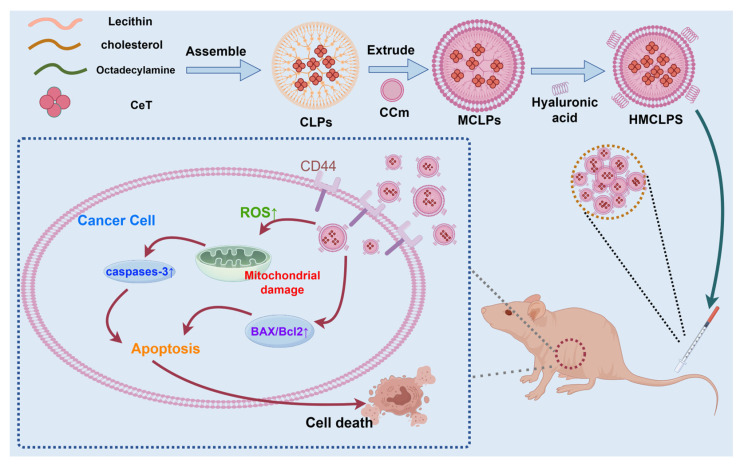
The design strategy of the nano-drug delivery platform HMCLPs.

In the present study, we successfully developed cationic liposomes loaded with CeT, modified them with HA to improve tumor targeting, and encapsulated them within cancer cell membranes. These hybrid membrane-coated liposome nanoparticles (HMCLPs) are designed to prolong the circulation time of CeT, reduce toxicity, and increase the selective accumulation of CeT at the tumor site (Scheme 1). Furthermore, HMCLPs exhibited reduced toxicity, increased targeting towards tumor cells, and played potent antitumor roles both in vitro and in vivo by inducing apoptosis in HCC cells. This strategy offers a promising new approach for the targeted treatment of HCC and provides new opportunities for the application of natural compounds such as CeT.

## 2. Materials and Methods

### 2.1. Chemical and Reagents

Celastrol was provided by Sigma-Aldrich Co. (St. Louis, MO, USA), while cholesterol, lecithin, stearylamine, and hyaluronic acid were obtained from Aladdin (Shanghai, China). Analytical reagents, such as methanol and ethanol, were sourced from Sinopharm Chemical Reagent Co., Ltd. (Shanghai, China). Cell culture materials, including fetal bovine serum (FBS) and Dulbecco’s modified Eagle’s medium (DMEM), were supplied by Gibco Inc. (Grand Island, NY, USA). The Cell Counting Kit-8 (CCK-8) and Membrane and Cytosol Protein Extraction Kit were purchased from Beyotime Biotechnology (Shanghai, China), and the Tunel apoptosis detection kit was obtained from Roche Pharmaceuticals Ltd. (Basel, Switzerland).

### 2.2. Cell Culture

Hep1-6, AML-12, and RAW 264.7 cell lines were procured from the Wuhan Cell Bank (Wuhan, China). Hep1-6, AML-12, and RAW 264.7 cells were cultured in DMEM supplemented with 10% FBS and 1% penicillin/streptomycin in a humidified incubator at 37 °C with 5% CO_2_.

### 2.3. Animals

Forty-six male Balb/c-nu mice (license No. HNUCM21-2406-12) (SPF, 18–22 g) were purchased from Slack Jingda Experimental Animal Company (Changsha, China) and received free food and water under a light–dark cycle. According to the “Guide for the Care and Use of Laboratory Animals” released by the National Institutes of Health, this experiment has been approved by the Institutional Ethical Committee on Animal Care and Experimentations of Hunan University of Chinese Medicine. After one week of adaptive feeding, 3 × 10^6^ Hep1-6 cells were subcutaneously injected. Cells were resuspended in cold PBS with a final volume of 100 μL for each injection. Tumor volumes were recorded at the indicated time points by measuring the tumors with a caliper and calculating the volumes with the formula L×W^2^/2 (L represents the length of the longer axis, and W represents the length of the shorter axis). After the tumor volume increased to 80–100 mm^3^, all mice were randomly divided into six groups (*n* = 8 mice per group): (1) the PBS group; (2) the free CeT group; (3) the CLPs group; (4) the HCLP group; (5) the MCLP group; and (6) the HMCLP group.

### 2.4. HPLC Assay

An Agilent C18 column (250 mm × 4.6 mm, 5 μm) was used in the HPLC experiment to detect Celastrol (CeT). The mobile phase consisted of methanol and 4% phosphoric acid (87:13, *v*/*v*) with a flow rate of 1.0 mL/min, the injection volume was 20 μL, the detection wavelength was set at 425 nm, and the column temperature was maintained at 30 °C. All reagents used were of HPLC grade. The HPLC method was validated for detecting CeT.

### 2.5. Preparation of CLPs

The nanoparticles were fabricated using the thin-film dispersion method, as described in previous studies [22]. Briefly, 40 mg of lecithin, 4 mg of stearylamine, 8 mg of cholesterol, 10 µL of Tween 80, and 4 mg of CeT were dissolved in 5 mL ethanol using a rotary vacuum evaporator for 1 h at 40 °C. Following evaporation, a lipid film formed on the bottom of the flask. The film was then hydrated with 5 mL of distilled water. The resulting suspension was emulsified in an ice-water bath for three minutes at 250 W using a sonicator. The emulsion was pre-cooled in an ice bath and subsequently sequentially filtered through microporous filters with pore sizes of 0.8, 0.45, and 0.22 µm before being extruded through a liposome extruder with a 0.1 µm filter. The product was dialyzed at 24 °C for 48 h to obtain the final product. For the preparation of fluorescent nanoparticles (NPs), C6 was substituted for CeT in the above procedures.

### 2.6. Preparation of the Cancer Cell Membrane

Hep1-6 cells were harvested from the culture dish using a cell scraper, washed with PBS, and the cell pellet was resuspended in a hypotonic lysis buffer containing membrane protein extraction reagent A and phenylmethylsulfonyl fluoride (PMSF) at a ratio of 100:1. The cells were lysed on ice for 30 min. Meanwhile, the lysate was sonicated using an ultrasonic cell disruptor for 3 min at 80–100 W. The lysate was then repeatedly frozen and thawed between −80 °C and 37 °C to further disrupt the cells. The lysate was centrifuged at 800 rpm for 10 min at 4 °C. The supernatant was collected and centrifuged again at 12,000 rpm for 30 min to isolate the cell membrane fragments. The collected cell membrane fragments were sequentially filtered through 0.45 μm and 0.22 μm filters. Finally, Hep1-6 cell membranes with particle sizes less than 200 nm were obtained, and membrane proteins were quantified using a BCA protein assay kit.

### 2.7. Preparation of HCLPs, MCLPs, HMCLPs

HCLPs: A total of 3 mL of CLP nanoparticles (1.0 mg/mL) was combined with 1 mL of HA vesicles (0.5 mg/mL). Electrostatic adsorption was employed to coat the hyaluronic acid onto the celastrol-loaded cationic liposomes. The electrostatic attraction between positive and negative charges ensured an even coating of hyaluronic acid on the surface of the celastrol-loaded cationic liposomes. MCLPs: A total of 3 mL of CLP nanoparticles (1.0 mg/mL) was combined with 1 mL of Hep1-6 cell membrane vesicles (0.5 mg/mL). The mixture was sonicated for 10 min at 250 W. Subsequently, the mixture was centrifuged at 4 °C and 14,000 rpm for 10 min to collect the cell membrane-wrapped MCLP nanoparticles, and the supernatant was discarded. The cell membrane-wrapped MCLP nanoparticles were resuspended in 3 mL of distilled water. HMCLPs: A total of 3 mL of CLP nanoparticles (1.0 mg/mL) was combined with 1 mL of Hep1-6 cell membrane vesicles (0.5 mg/mL) and 1 mL of HA vesicles (0.5 mg/mL). The mixture was ultrasonicated in an ice bath at 250 W for three minutes. The mixture was filtered sequentially through 0.8, 0.45, and 0.22 µm microporous filters, followed by extrusion through a 0.1 µm liposome extruder. The resulting product was dialyzed at 24 °C for 48 h to obtain the final product.

### 2.8. The Encapsulation Efficiency (EE) and Drug-Loading Efficiency (DL) of NPs

The concentration of CeT in the nanoparticles was quantified using an HPLC assay. A 100 μL aliquot of the nanoparticle suspension was transferred to a 1.5 mL centrifuge tube, and 900 μL of ethanol was added to disrupt emulsification. The supernatant was collected after centrifugation at 1000 rpm and filtered through a 0.22 μm microporous membrane. The filtrate was analyzed by HPLC to determine the free CeT concentration. Drug loading (DL) was calculated using Formula (1), and encapsulation efficiency (EE) was determined using Formula (2):

(1) $$DL\%=\frac{W_{T}-W_{F}}{W_{E}}*100\%$$

(2) $$EE\%=\frac{W_{T}-W_{F}}{W_{T}}*100\%$$

In the formula, W_T_ represents the total drug in the formulation, W_F_ is the free drug in the filtrate, and W_E_ is the total weight of excipients used in the formulation.

### 2.9. Characterization of the Prepared Nanoparticles

The particle size, polydispersity index (PDI), and zeta potential of the nanoparticles were assessed using a Malvern ZS90 Zetasizer (Malvern, Worcestershire, UK). Nanoparticle stability was evaluated with a JEM-1011 transmission electron microscope (TEM) (JEOL, Ltd., Tokyo, Japan). Protein characterization on the nanoparticles was conducted using SDS-PAGE.

### 2.10. In Vitro Release of CeT

The dialysis method was used to detect the drug release profiles of CLPs, HCLPs, MCLPs, HMCLPs, and free CeT. A 2 mL aliquot of each suspension (containing 1 mg CeT) was transferred into separate dialysis bags and immersed in 50 mL of release buffer (containing 0.1% *w*/*v* Tween 80, pH 5.5). The setups were shaken at 100 rpm and 37 °C. At time points 0, 1, 2, 4, 8, 16, 24, 36, and 48 h, 1 mL of the external medium was removed, replaced with an equal volume of fresh, pre-warmed medium, and the concentration of CeT in the release medium was analyzed using HPLC.

### 2.11. Cellular Uptake Assays In Vitro

Cellular uptake of nanoparticles in vitro was evaluated using Hep1-6 cancer cells, AML-12 normal mouse hepatocytes, and RAW264.7 macrophages. The cancer cell-HA hybrid membrane-camouflaged lipid nanoparticles (HMCLPs) were labeled with C6. Cellular uptake was evaluated using fluorescence microscopy (Leica, Germany) and FACScanto flow cytometry (BD Biosciences, Franklin Lakes, NJ, USA). For fluorescence observation, Hep1-6 cancer cells, AML-12 normal mouse hepatocytes, and RAW264.7 macrophages were, respectively, seeded into glass-bottom culture dishes at a density of 5 × 10^4^ cells per well 24 h prior to the experiment. Cells were then incubated with C6-labeled CLPs, HCLPs, MCLPs, or HMCLPs at 37 °C for 2 h. Subsequently, the C6-labeled nanoparticles were removed, and the cells were fixed with 4% paraformaldehyde and then stained with Hoechst 33,342 for 15 min. Finally, the cells were washed three times with PBS and observed under a fluorescence microscope. For flow cytometric analysis, the cells were incubated with C6-tagged CLPs, HCLPs, MCLPs, or HMCLPs for 2 h at 37 °C. The C6-tagged nanoparticles were removed, and the cells were digested with trypsin, collected by centrifugation, and washed three times with PBS. Finally, the cells were resuspended in PBS for flow cytometric analysis.

### 2.12. Cytotoxicity and Apoptosis Studies on Nanoparticles In Vitro

The CCK-8 assay was employed to assess the effects of free CeT, CLPs, HCLPs, MCLPs, and HMCLPs on the viability of Hep1-6 and AML-12 cells. Hep1-6 and AML-12 cells were suspended in medium at a concentration of 3 × 10^4^ cells/mL, and 100 μL of this suspension was added to each well of a 96-well plate and incubated overnight. Following 24 h of treatment, 10 μL of CCK-8 reagent was introduced to each well, and the plates were incubated for an additional hour. Optical density (OD) was measured at 450 nm using a microplate reader. Cell viability was calculated using the provided Formula (3):

(3) $$survival\;rate\left(\%\right)=\frac{\left(As-Ab\right)}{\left(Ac-Ab\right)}*100\%$$

In the formula, A**_s_** is the OD of the experimental group (cell-containing culture medium, CCK-8 reagent, NPs value). A**_c_** is the OD value of the control group (culture medium containing cells, CCK-8 reagent, and no NPs), and A**_b_** is the OD value of the blank group (culture medium containing cells and NPs, CCK-8 reagent).

### 2.13. Apoptosis Analysis

Hep1-6 cells were cultures in 6-well plates at a density of 2 × 10^6^ cells/m. The cells were then co-incubated with CeT nanoparticles (various formulations, each at an equivalent CeT concentration of 1.0 μM) for 24 h. Subsequently, the cells were collected after trypsin digestion, stained with annexin V-FITC and propidium iodide (PI), and resuspended in 500 μL of binding buffer. The samples were analyzed using flow cytometry.

### 2.14. In Vivo Homologous Targeting and Biodistribution of HMCLPs

To evaluate the homologous cell membrane targeting capability of HMCLPs, tumor-bearing mice were randomly assigned to PBS, Free DIR, DLPs, HDLPs, MDLPs, and HMDLPs groups (three mice in each group), and DIR-labeled nanoparticles were administered via the tail vein, except for the PBS group, the DIR concentration in each group of nanoparticles was 10 μL/mL. After 24 h of administration, the mice were anesthetized with isoflurane, and the biodistribution of the DIR-labeled nanoparticles was assessed using a real-time in vivo fluorescent imaging system (IVIS Lumina XRMS series instrument, PerkinElmer, Waltham, MA, USA) with an excitation wavelength of 748 nm. The fluorescence distribution in excised tissues was also examined.

### 2.15. Antitumor Activity of Nanoparticles In Vivo

Each group of mice received 7 intravenous injections within 14 days. Except for the PBS group, the CeT dose in the nanoparticles for each group was 0.2 mg/kg. Every two days, the researchers recorded the body weight and tumor volume. Mice were euthanized two days following the final injection, and the tumors were removed, weighed, and examined with H&E staining, Ki67 immunohistochemistry (IHC), and Tunel assays. Mouse blood was then collected for biochemical index detection, including CRE, BUN, ALT, AST, and ALP, which are biochemical indicators of liver and kidney damage.

### 2.16. Statistical Analysis

A one-way *ANOVA* followed by the Bonferroni test was used for multiple group comparisons with GraphPad Prism 8.0. A two-tailed Student’s *t*-test was used for comparisons between two groups. *p* < 0.05 was considered a significant difference.

## 3. Results

### 3.1. Fabrication and Characterization of Nanoparticles

**Figure 1:**
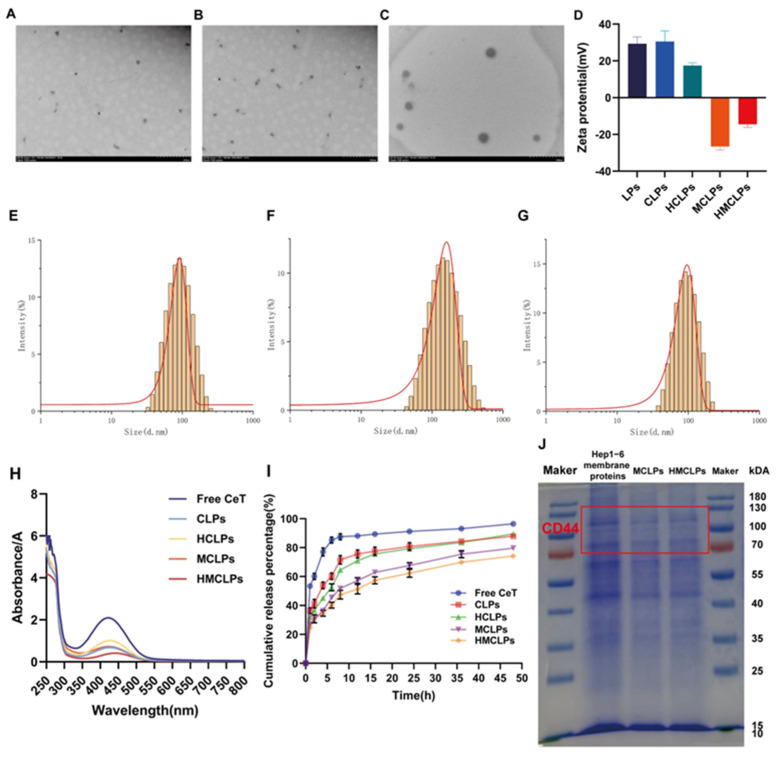
Characterization of HMCLPs. (**A**) TEM micrographs of LPs, ×20 k; (**B**) TEM micrographs of CLPs; (**C**) TEM micrographs of HMCLPs. (**D**) Zeta potential of HMCLPs. (**E**) Size distribution of LPs. (**F**) Size distribution of CLPs. (**G**) Size distribution of HMCLPs. (**H**) Changes in UV absorption wavelength of nanoparticles. (**I**) In vitro release curves of CeT from free CeT, LPs, CLPs, HCLPs, and MCLPs. (**J**) Gel electrophoresis analysis of HMCLPs, MCLPs nanoparticles, and Hep1-6 cell membrane vesicles.

HMCLPs were prepared using the emulsification method (see Figures S1–S3 and Tables S1–S4). To ensure prolonged circulation and effective tumor targeting, HMCLPs were covered with Hep1-6 cell membranes. The drug loading (DL) of CeT in HMCLPs was 3.7%. The transmission electron microscopy (TEM) images of LPs, CLPs, and HMCLPs (Figure 1A–C) showed that HMCLPs were spherical. The formulation without drug loading appears milky white (Figure S3), while HMCLPs and MCLPs exhibit core–shell structures, which is different from LPs, CLPs, and HCLPs after being covered by hybrid membranes. The DLS analysis showed that its particle dispersion index (PDI) is small and has a relatively uniform particle size distribution. The particle sizes of LPs, CLPs, HCLPs, MCLPs, and HMCLPs were 80.61 ± 2.14 nm, 84.47 ± 3.14 nm, 110.03 ± 4.41 nm, 109.3 ± 4.94 nm, and 128.5 ± 5.84 nm, respectively, with zeta potentials of 33.6, 37.2, 17.6, −28.6, and −16.4 mV, indicating good stability (Figure 1D–H, Table S5). The increase in particle size and the decrease in zeta potential are likely due to the negatively charged hybrid membrane, which has an approximate thickness of 10–20 nm. Drug release studies in PBS containing 0.1% *w*/*v* Tween 80 (Figure 1I) demonstrated a two-phase release profile: an initial burst within 4 h, followed by a slower release from 1 to 48 h. The cumulative release percentages of CeT at 24 h were 92.4% for free CeT, 83.6% for CLPs, 80.3% for HCLPs, 66.3% for MCLPs, and 60.6% for HMCLPs. At 48 h, the release percentages were 96.1%, 89.4%, 88.2%, 80.2%, and 76.3%, respectively. The slower release observed for HCLPs, MCLPs, and HMCLPs at earlier time points is likely attributed to the HA membrane camouflage. SDS-PAGE analysis (Figure 1J) confirmed that HMCLPs and MCLPs retained characteristic proteins from the Hep1-6 membrane, such as CD44. These results indicate the successful establishment of HMCLPs as a nano-drug carrier platform with a particle size of 140 nm, which is wrapped with HA and Hep1-6 cell membranes and capable of sustaining drug release of sustaining drug release.

### 3.2. HMCLPs Can Escape Immune Cell and Lysosomal Clearance

**Figure 2:**
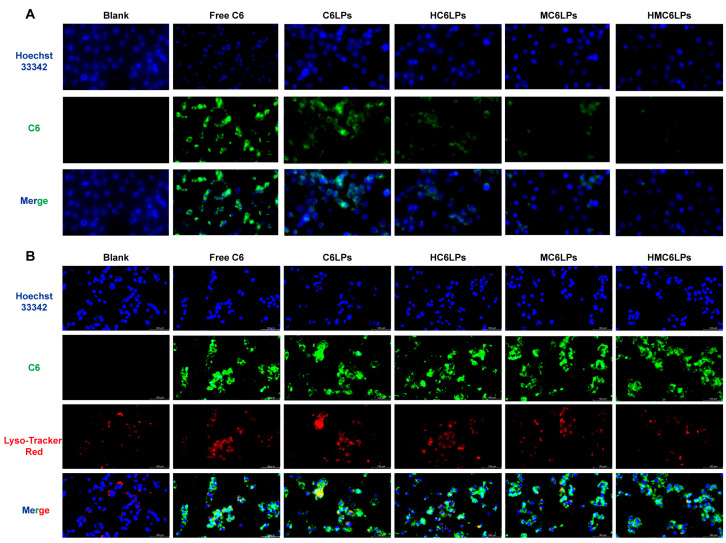
Escape ability of various nanoparticles. (**A**) Confocal microscopy image of RAW264.7 cell uptake, ×100; (**B**) confocal microscopy image of nanoparticle lysosomal escape, ×100.

To evaluate uniform tumor targeting and prolonged circulation, nanoparticles were assessed using cellular uptake assays. The delivery efficiency of nanoparticles primarily depends on their ability to evade clearance by the mononuclear phagocyte system. RAW 264.7 is a major representative of the mononuclear phagocyte system. The effects of cancer cell membrane camouflage and HA modification on cellular uptake by RAW 264.7 were detected using fluorescence microscopy and quantitative FACS analysis to assess immune evasion. Nanoparticles were labeled with the hydrophobic fluorescent dye C6. LCSM images showed that free C6 and CLPs were extensively internalized by macrophages, with strong green fluorescence (Figure 2A). However, green fluorescence signals for HCLPs, MCLPs, and HMCLPs in RAW 264.7 cells were weak. FACS analysis revealed that the cellular internalization rates of HCLPs, MCLPs, and HMCLPs were significantly lower compared to CLPs, indicating that membrane camouflage and HA modification significantly reduce macrophage internalization.

In vivo, nanomedicines typically enter cells via endocytosis, move into endosomes, and are subsequently transported to lysosomes. Lysosomes are acidic organelles containing various digestive enzymes that break down and degrade foreign substances. If nanomedicines fail to escape lysosomes, they may be degraded, leading to drug inactivation or loss of therapeutic efficacy. Hence, lysosomal escape is crucial for nanomedicine effectiveness. Lysosomes were labeled with Lyso-Tracker Red, and nanoparticles were labeled with C6 to verify lysosomal escape ability. Results showed that modifying the nanoparticle surface enhances lysosomal escape, thereby preventing drug degradation and improving delivery efficiency (Figure 2B and Figure S4).

These results demonstrate that HMCLPs effectively evade immune system clearance and lysosomal degradation, enabling efficient drug delivery to the target site. Compared to free drugs and unmodified liposomes, their escape ability is significantly enhanced.

### 3.3. HMCLP Can Specifically Target HCC Cells

When used as drug carriers, nanomaterials must be efficiently internalized by target cells to ensure effective drug delivery and therapeutic outcomes. Insufficient cellular uptake can impair drug delivery efficiency and reduce therapeutic efficacy. Ideally, nanomaterials should exhibit high targeting specificity, meaning they are effectively taken up by specific cell types (e.g., cancer cells) while showing reduced uptake by normal cells. The extent of cellular uptake directly influences targeting efficiency and treatment specificity.

**Figure 3:**
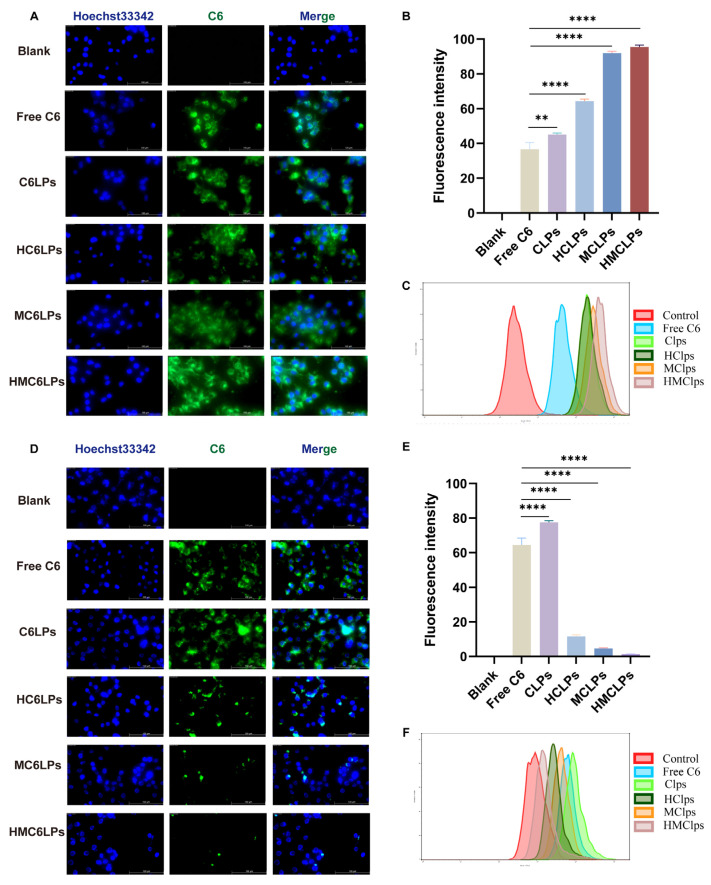
Cellular uptake and distribution after incubation with various nanoparticles. (**A**) Confocal microscopy image of nanoparticle uptake by Hep1-6 cells, ×100; (**B**) statistical analysis of nanoparticle uptake by Hep1-6 cells; (**C**) flow cytometry was used to detect the fluorescence intensity of Hep1-6 cells after taking up nanoparticles; (**D**) confocal microscopy image of nanoparticle uptake by AML-12 cells, ×100; (**E**) statistical analysis of nanoparticle uptake by AML-12 cells; (**F**) flow cytometry was used to detect the fluorescence intensity of AML-12 cells after taking up nanoparticles. Mean ± SD; *n* = 3; ** *p* < 0.01, and **** *p* < 0.0001 compared to free C6 group.

To assess the impact of membrane camouflage and HA modification on cellular uptake, nanoparticles were labeled with the hydrophobic fluorescent dye C6. LCSM images revealed that HCLPs, MCLPs, and HMCLPs were internalized more readily by Hep1-6 cells compared to CLPs, exhibiting stronger green fluorescence signals (Figure 3A). Additionally, fluorescence signals from HMCLPs, HCLPs, and MCLPs in Hep1-6 cells were higher than those from CLPs and free C6 (Figure 3B,C), confirming that both cancer cell membrane camouflage and HA modification enhance cellular uptake, with HMCLPs showing the most significant effect.

Conversely, LCSM images indicated that the internalization of HCLPs, MCLPs, and HMCLPs by AML-12 cells was lower compared to CLPs and free C6 (Figure 3D), suggesting reduced uptake by normal liver cells and effective targeting of tumor cells (Figure 3E,F). Results from various incubation times supported this conclusion (Figure S5). The data demonstrate that HMCLPs exhibit substantially higher uptake in liver cancer cells relative to normal liver cells, showcasing excellent targeting and specificity for liver cancer cells. This targeted approach helps reduce the required drug dose, minimizes side effects, and improves drug bioavailability.

### 3.4. HMCLPs Inhibit the Proliferation of HCC Cells

**Figure 4:**
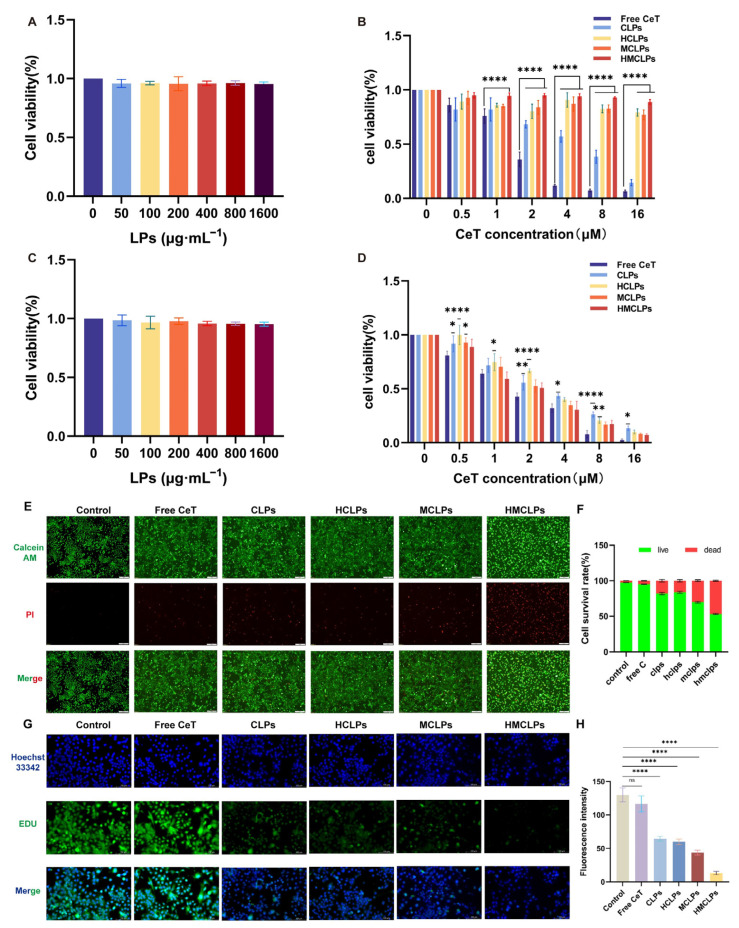
In vitro antitumor activity of various nanoparticles. (**A**) The effect of undrug-loaded liposomes on AML-12 cell viability. (**B**) The effect of CeT-loaded liposomes on AML-12 cell viability compared to the free CeT group. (**C**) The effect of undrug-loaded liposomes on Hep1-6 cell viability. (**D**) The effect of CeT-loaded liposomes on Hep1-6 cell viability compared to the free CeT group. (**E**) Calcein AM staining results, ×50; (**F**) Calcein AM statistical analysis. (**G**) Edu fluorescence analysis, ×100. (**H**) Edu fluorescence intensity statistical results. Mean ± SD; *n* = 3; The ns (*p* > 0.05), * *p* < 0.05, ** *p* < 0.01, and **** *p* < 0.0001, (**B**,**D**) compared to free CeT group, (**H**) compared to control group.

Nanomedicines exhibit significant cytotoxicity and can directly kill cancer cells. This cytotoxicity is achieved by precisely delivering drugs to cancer cells via nanodrug carriers, thereby minimizing damage to normal cells. Compared to traditional chemotherapeutic agents, nanomedicines can enhance therapeutic efficacy by increasing drug concentration at the tumor site through surface modification or specific targeting ligands. We employed the CCK-8 assay to assess the in vitro cytotoxicity of empty and drug-loaded nanoparticles against Hep1-6 and AML-12 cells. After 24 and 48 h of incubation, free drugs and unmodified liposomes exhibited substantial toxicity to AML-12 cells, whereas the toxicity of modified liposomes was notably reduced (Figure 4A,B and Figure S6A). All formulations exhibited dose-dependent inhibitory activity against Hep1-6 cells (Figure 4C,D and Figure S6B). In addition, the blank carrier LPs showed no obvious toxicity to normal liver cells and liver cancer cells within the concentration range of 0–1600 μg/mL. Among the various drug preparations, HMCLP exhibited the highest cytotoxicity against Hep1-6 cells and showed the best safety profile toward normal cells at all tested concentrations. The enhanced cytotoxicity indicates that tumor-targeted nanoparticles can effectively deliver multiple drug molecules into Hep1-6 cells simultaneously while reducing uptake by normal hepatocytes. Calcein AM/PI staining and EDU assays revealed that HMCLPs had a significant killing effect and inhibitory impact on cell proliferation compared with free drugs and unmodified nanoparticles (Figure 4E,H). Combining Calcein AM/PI staining with EDU detection enables simultaneous evaluation of cell killing and proliferation inhibition, offering a comprehensive assessment of nanoparticle efficacy. This multidimensional approach enhances the understanding of how nanoparticles induce cell apoptosis.

### 3.5. HMCLPs Induce Apoptosis in HCC Cells

**Figure 5:**
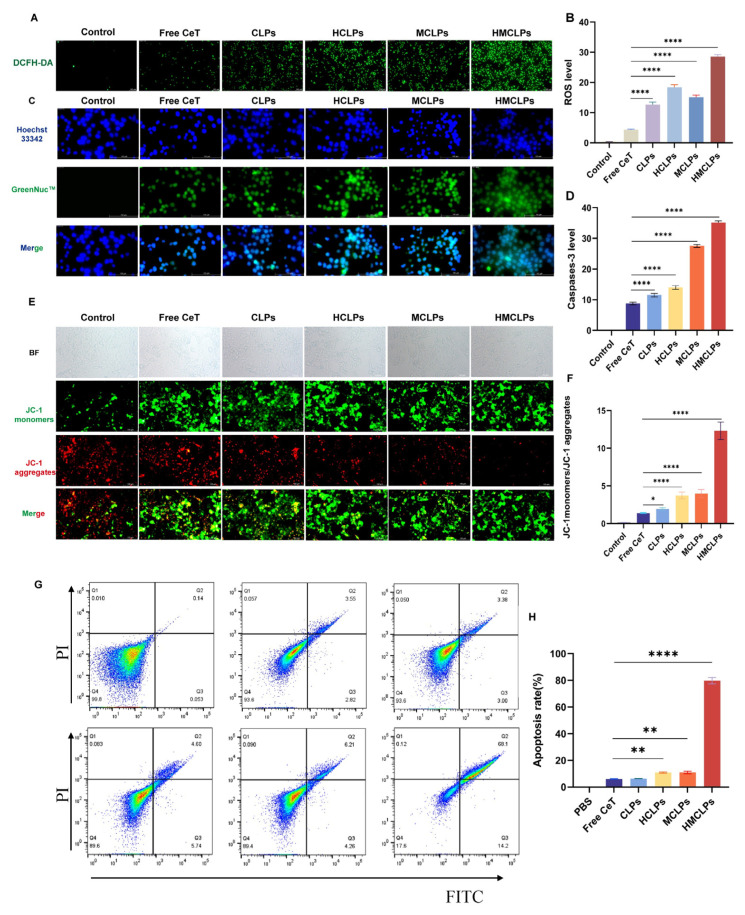
The capacity of several nanoparticles to trigger apoptosis in HCC cells. (**A**) Nanoparticles can stimulate an increase in ROS in Hep1-6 cells, ×50; (**B**) ROS fluorescence statistical results; (**C**) nanoparticles can increase caspases-3 in living cells, ×100; (**D**) fluorescence statistical results; (**E**) nanoparticles can change mitochondrial membrane potential, ×100; (**F**) mitochondrial membrane potential statistical results; (**G**) apoptosis flow cytometry; (**H**) flow cytometry statistical results. Mean ± SD; *n* = 3; * *p* < 0.05, ** *p* < 0.01, and **** *p* < 0.0001 compared to free CeT group.

Apoptosis is a regulated cell death process crucial for maintaining tissue homeostasis and eliminating damaged cells. Reactive oxygen species (ROS) induce apoptosis through various mechanisms, including oxidative damage, activation of signaling pathways, and changes in mitochondrial function. Detection with DCFH-DA and GreenNuc^TM^ fluorescent probes (Beyotime Biotechnology, Shanghai, China) demonstrated that HMCLPs elevate ROS levels and caspase-3 activity (Figure 5A,D). Excessive ROS production can compromise mitochondrial membrane integrity, causing proton leakage and dysfunction of the mitochondrial respiratory chain, which leads to a reduction in mitochondrial membrane potential (Figure 5E,F). A decrease in mitochondrial membrane potential is a key marker of apoptosis. During apoptosis, caspase-3 cleaves several critical proteins, resulting in irreversible cellular damage. Caspase-3 activation typically indicates the onset of irreversible apoptosis. Annexin V-FITC/PI double staining showed that the proportion of early and late apoptotic cells was the highest in the HMCLPs group, with an apoptotic rate of 68.2%, which was significantly higher than that of the other groups. When the cell nuclei were stained with Hoechst 33342, chromatin condensation and nuclear fragmenta-tion were observed under a fluorescence microscope, which are characteristic morphological changes during cell apoptosis (Figure 5G,H and Figure S7). Western blot experiments showed that, compared with the control group, Bax expression in the MCLPs and HMCLPs groups was markedly elevated, while Bcl-2 expression was notably reduced, resulting in a pronounced increase in the Bax/Bcl-2 ratio in these two groups. This shift indicates the activation of apoptotic signals, along with an upregulation of cleaved-caspase-3 (activated caspase-3), suggesting that drug-loaded nanoparticles can induce apoptosis through a caspase-3-dependent pathway (Figure S8). These findings suggest that HMCLPs induce mitochondrial damage, alter the Bax/Bcl-2 ratio, and activate caspase-3 by increasing intracellular ROS levels, thereby promoting cancer cell apoptosis and demonstrating superior antitumor efficacy compared to free drugs and unmodified nanomedicines.

### 3.6. In Vivo Biodistribution of Homologous-Targeting Nanoparticles

**Figure 6:**
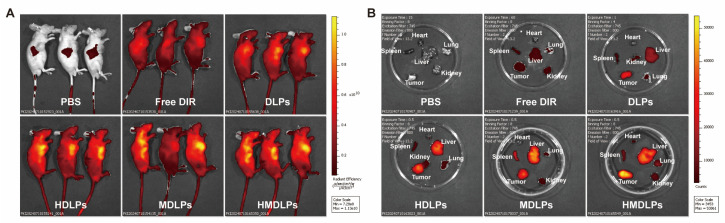
In vivo biodistribution and ex vivo organ fluorescence imaging of various nanoparticles. (**A**) Fluorescence images of tumor-bearing Balb/c-nu mice at different time points after intravenous injection of various DIR-labeled preparations; (**B**) fluorescence images of excised tissues.

DIR-labeled nanoparticles were synthesized. Following tail vein injection, the distribution and accumulation of nanoparticles at the tumor site in tumor-bearing mice were monitored using a real-time in vivo fluorescence imaging system. As shown in Figure 6A, 24 h after injection, the strongest fluorescence signal was observed in the tumor tissue of DIR-labeled HMCLPs, followed by HCLPs and MCLPs, with the CLP-treated tumor tissue exhibiting the weakest fluorescence. This suggests that HMCLPs, modified with cancer cell membrane camouflage and HA, effectively target tumors and exhibit prolonged circulation, leading to greater accumulation in tumor tissues. Concerning other tissues (Figure 6B), the HMCLPs group displayed the highest fluorescence among all test groups at the tumor site. Therefore, HMCLPs demonstrate superior antitumor effects compared to CLPs, HCLPs, and MCLPs.

### 3.7. In Vivo Antitumor Effect of HMCLPs in Subcutaneous HCC Model

**Figure 7:**
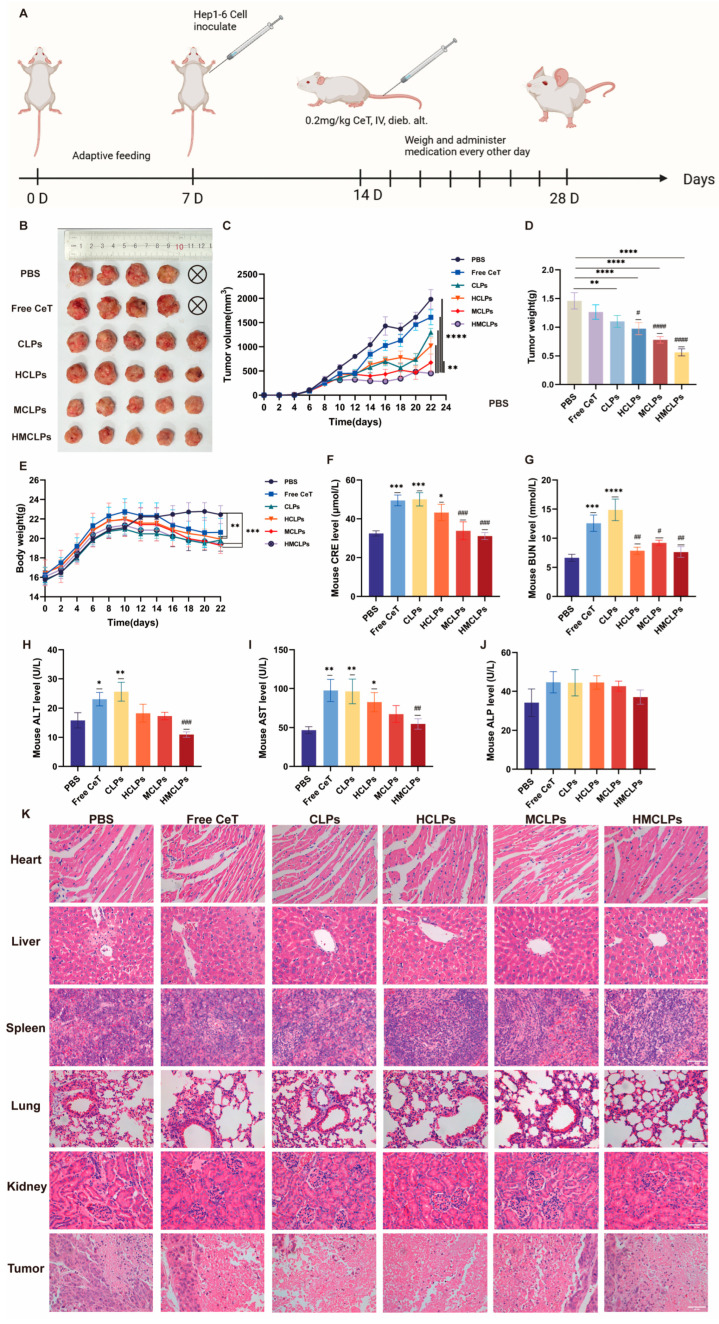
In vivo antitumor activity of tumor-bearing Balb/c-nu mice treated with various nanoparticles. (**A**) Flow chart of in vivo antitumor technology; (**B**) photos of tumor size; (**C**) tumor volume; (**D**) tumor weight; (**E**) body weight changes; (**F**) results of CRE levels; (**G**) results of BUN levels; (**H**) results of ALT levels; (**I**) results of AST levels; (**J**) results of ALP levels; (**K**) H&E staining results of various organs, ×400. Mean ± SD; *n* = 5; * *p* < 0.05, ** *p* < 0.01, *** *p* < 0.001, and **** *p* < 0.0001 compared to PBS group; ^#^ *p* < 0.05, ^##^ *p* < 0.01, ^###^ *p* < 0.001, and ^####^ *p* < 0.0001 compared to free CeT group.

The antitumor efficacy of each nanoparticle group was evaluated in Hep1-6 Balb/c-nu tumor-bearing mice. Results revealed sustained tumor growth in mice treated with PBS and free CeT, likely due to inadequate retention of CeT at the tumor area (Figure 7B). Tumor growth was also observed in mice treated with CLPs; however, it was significantly suppressed in mice treated with HCLPs, MCLPs, and HMCLPs. Among these, the HMCLPs group exhibited the slowest tumor growth, the smallest tumor size, and the lightest tumor weight by the end of the treatment (Figure 7B–E), suggesting that HMCLPs and other modified nanoparticles provided superior antitumor effects. In addition, the fluctuations in the body weight of tumor-bearing mice during and after treatment may be due to the lipid-lowering and weight-loss effects of CeT. Biochemical tests were conducted on mice’s serum to detect liver and kidney injury-related markers such as CRE, BUN, AST, ALT, and ALP. The results showed that compared with free CeT and unmodified nanoparticles, HMCLPs and modified nanoparticles did not cause significant hepatotoxicity or renal toxicity, indicating good biocompatibility and safety (Figure 7F–J). Furthermore, H&E staining results showed no significant differences among the organs from mice in each group, confirming that the modified nanoparticles did not induce notable toxic side effects (Figure 7K). These findings highlighted that HMCLPs exhibited potent antitumor activity with minimal off-target toxicity, suggesting effective targeting and a low likelihood of adverse effects on non-target organs.

### 3.8. HMCLPs Trigger Apoptosis to Impede the Progression of HCC

**Figure 8:**
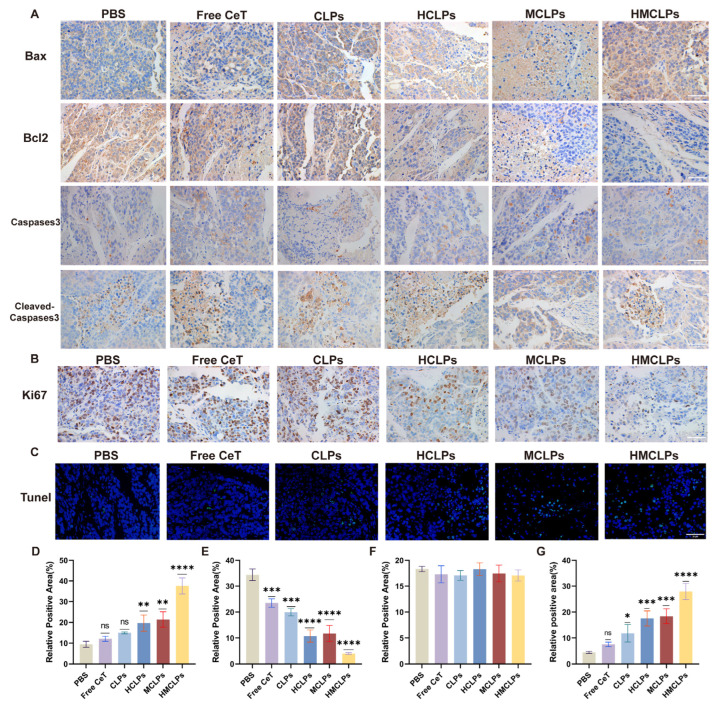
Immunohistochemical and Tunel analyses of tumor tissues treated with various formulations. (**A**) Tumor immunohistochemistry, ×400; (**B**) Ki67 staining, ×400; (**C**) Tunel assay, ×400; (**D**) Bax staining; (**E**) Bcl-2 staining; (**F**) Caspase-3 staining; (**G**) Cleaved-caspase-3 staining. Mean ± SD, *n* = 3. The ns (*p* > 0.05), * *p* < 0.05, ** *p* < 0.01, *** *p* < 0.001, **** *p* < 0.0001 vs. PBS group.

Immunohistochemistry revealed that HMCLPs elevated caspase-3 cleavage fragments and altered the Bax/Bcl-2 ratio, promoting apoptosis and enhancing the antitumor effect (Figure 8A,D–G). Elevated Ki67 expression in tumor tissues was typically associated with rapidly proliferating malignant tumors, reflecting the high proliferative activity of tumor cells. A higher Ki67 index correlated with more rapid tumor growth and increased invasiveness. Ki67 staining results (Figure 8B) demonstrated that the modified nanoparticles effectively inhibited cell proliferation. The in situ Tunel assay (Figure 8C) showed minimal green fluorescence in tumors from the PBS treatment group, indicating a lack of apoptosis. In contrast, tumors from the HMCLPs treatment group exhibited the most intense green fluorescence, reflecting the highest proportion of apoptotic tumor cells and the most effective treatment. Ki67 and Tunel assays assessed proliferation and apoptosis, respectively. By comparing these results, we can evaluate the balance between cell proliferation and apoptosis during treatment. The reduced number of Ki67-positive cells and increased number of Tunel-positive cells suggested that the treatment effectively suppressed tumor cell proliferation and induced apoptosis.

## 4. Discussion

CeT is a pentacyclic triterpenoid compound with various biological activities, including anti-inflammatory, anti-cancer, antioxidant, lipid-lowering, and immunosuppressive effects. However, the application of CeT is limited by its toxicity and poor solubility. The present study proposes a novel hybrid nanoparticle platform, HMCLP, aimed at improving the pharmacological effects of CeT while minimizing its toxicity. Our results demonstrated that HMCLPs dramatically reduce the hepatotoxicity and nephrotoxicity of CeT, which is the major limitation of its application. At present, biomimetic systems, such as HMCLPs, were developed successfully in this study and have significant value in the field of drug delivery. In recent years, various research groups have explored numerous strategies to create biomimetic nanosystems that can improve drug targeting and reduce off-target effects. The HMCLPs designed here used a cationic liposome core modified with HA and camouflaged by cancer cell membranes to target tumor cells through multiple mechanisms, including the homing effects and CD44 receptor-mediated endocytosis, making HMCLPs an effective strategy for tumor targeting. Furthermore, cancer cell membrane coatings allow nanoparticles to interact with the tumor microenvironment, facilitating transport across biological barriers [23,24,25]. Enzymes such as hyaluronidases are overexpressed in the cancer microenvironment, degrading HA into smaller fragments. These fragments can further strengthen the binding of HA to CD44 receptors and promote receptor aggregation and activation, thereby enhancing the internalization of HA-encapsulated drug delivery systems [14,26,27,28]. The degradation products of HA can also lead to alterations in the tumor microenvironment. For instance, the degradation products of HA can increase the permeability of the extracellular matrix (ECM) and tumor vasculature, making it easier for drugs to penetrate tumor tissues.

Previous studies have demonstrated that the antitumor effect of HMCLPs is mainly attributed to their ability to increase reactive ROS levels, which leads to mitochondrial membrane disruption, increased permeability, and subsequently decreased mitochondrial membrane potential [29,30,31], and activates downstream effector caspases, such as caspase-3, thereby initiating a proteolytic cascade [29,32,33]. Additionally, HMCLPs can initiate apoptosis signaling pathways, such as the Bax/Bcl-2 pathways, by causing oxidative damage to cellular lipids, proteins, and DNA, ultimately inducing tumor cell death. However, these significant therapeutic efficacies largely depend on effective and targeted delivery to the tumor site.

The lack of targeted delivery is a major limitation of many conventional drugs. This study emphasizes the importance of targeted delivery in cancer therapy, as evidenced by the reduced uptake of HMCLPs in normal liver cells (AML-12) and increased accumulation of HMCLPs in tumor cells (Hep1-6). This selective targeting is further supported by the ability of HMCLPs to evade the detection by the mononuclear phagocyte system (MPS), preventing premature clearance from the body and improving drug delivery efficiency [34]. By effectively avoiding rapid uptake by macrophages, these particles can maintain higher concentrations in the systemic circulation, thereby promoting the delivery of therapeutic agents to the tumor sites. This dual functionality makes HMCLPs superior to single-targeted delivery systems, as the latter typically face limitations such as shortened circulation time and poor targeting efficiency [35,36]. The multi-targeting approach shows enhanced targeting capabilities and reduced off-target abilities compared with the single-target delivery systems [37]. Although a series of promising results have been achieved, this study still requires further refinement to fully realize its potential impact.

While HMCLPs (Hybrid Multi-Component Lipid Particles) have demonstrated superior antitumor activity and improved targeting capabilities compared to single-modification nanoparticles, it is essential to thoroughly assess their safety, efficacy, and long-term effects. A comprehensive understanding of their pharmacokinetics, biodistribution, and potential off-target effects is needed to ensure clinical safety and effectiveness. Such studies will not only confirm the utility of HMCLPs as a novel anti-cancer strategy but also help refine their design to maximize therapeutic outcomes.

## 5. Conclusions

In this study, we successfully developed a novel nanobiomimetic platform, HMCLPs, to encapsulate CeT for targeted HCC therapy. By utilizing homologous cell membranes and HA, we achieved enhanced targeting capabilities and controlled drug release, overcoming CeT’s previous limitations of toxicity and poor targeting. The advantages of this approach include improved bioavailability, enhanced accumulation of ROS, and reduced systemic toxicity. The use of HMCLPs not only improves CeT’s therapeutic efficacy but also mitigates its drawbacks, offering a safer and more potent treatment option for HCC. Mechanistically, CeT-loaded HMCLPs increase ROS production, induce mitochondrial damage, and activate caspase-3, leading to tumor cell apoptosis, which provides a promising strategy for future cancer treatment.

## Data Availability

The data that support the findings of this study are available from the corresponding authors upon reasonable request.

## Supplementary Materials

The following supporting information can be downloaded at https://www.mdpi.com/article/10.3390/cells13211819/s1. Figure S1. Establishment of an in vitro HPLC method for detecting CeT; Figure S2. Selection of lipid nanoparticle preparation process; Figure S3. Appearance of lipid nanoparticles; Figure S4. Results of lysosomal uptake of nanoparticles at different times; Figure S5. Nanoparticle cell uptake results at different times; Figure S6. Effects of different nanoparticles on the viability of AML-12 and Hep1-6 cells at 48 h; Figure S7. Hoechst 33 342 staining results of cells after treatment with different nanoparticles; Figure S8 Western blot analysis of apoptotic markers in different treatment groups; Table S1. Investigation on the precision of HPLC detection method for CeT; Table S2. Stability study of HPLC detection method for CeT; Table S3. Repeatability study of HPLC detection method for CeT; Table S4. Investigation on sample recovery rate of HPLC detection method for CeT; Table S5. Particle size, zeta potential, and encapsulation efficiency of different samples.
