# Correction: He et al. Celastrol-Loaded Hyaluronic Acid/Cancer Cell Membrane Lipid Nanoparticles for Targeted Hepatocellular Carcinoma Prevention. *Cells* 2024, *13*, 1819

**DOI:** 10.3390/cells15050426

**Published:** 2026-02-28

**Authors:** Peng He, Manshu Zou, Chanjuan Zhang, Yaning Shi, Li Qin

**Affiliations:** 1 Laboratory of Stem Cell Regulation and Application of Traditional Chinese Medicine, School of Pharmacy, Hunan University of Chinese Medicine, Changsha 410208, China; 20223702@stu.hnucm.edu.cn (P.H.); zcj@hnucm.edu.cn (C.Z.); syn@hnucm.edu.cn (Y.S.); 2 Science and Technology Innovation Center, Hunan University of Chinese Medicine, Changsha 410208, China; zoumanshu@hnucm.edu.cn

In the original publication [1], there were errors in the representative images shown in Figure 2A (C6LPs), Figure 7K (HCLPs heart H&E staining), Supplementary Figure S5A (C6LPs), and Supplementary Figure S5C (MC6LPs). The authors sincerely apologize for these errors, which resulted from oversights during image selection and figure assembly.

Upon careful re-examination of the original data, we found that the image used for the C6LPs group in Figure 2A was mistakenly taken from the image set belonging to the Free CeT group shown in Figure 3D during figure preparation. In addition, during the compilation of representative images for Figure 7K (HCLPs heart), an image intended for the HCLPs group was incorrectly copied from the CLPs group. Furthermore, incorrect images were inadvertently included in Supplementary Figure S5A (C6LPs) and S5C (MC6LPs) due to errors during the organization of multi-time-point cellular uptake experiment images. The correct original images from the respective experimental batches have been retrieved and used to replace the erroneous ones. The corrected figures now accurately represent the intended experimental data. We deeply regret these oversights and wish to confirm that these corrections do not affect the reported results or conclusions of the study, as the experimental analyses and findings remain unchanged.

**Figure 2:**
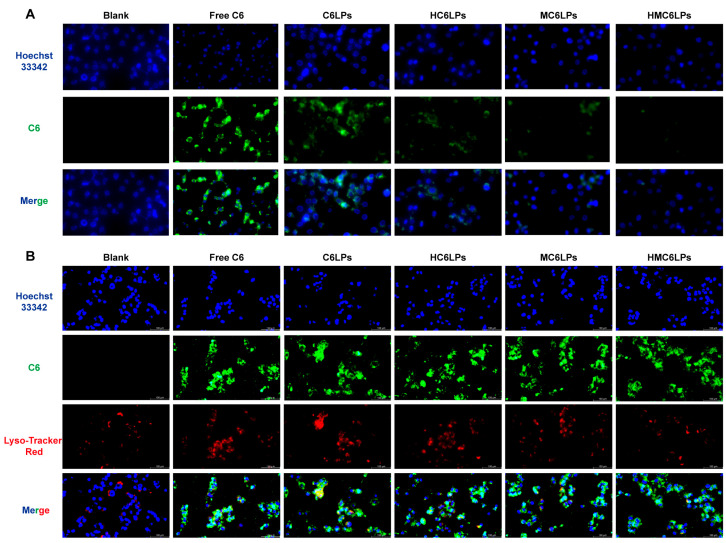
Escape ability of various nanoparticles. (**A**) Confocal microscopy image of RAW264.7 cell uptake, ×100; (**B**) confocal microscopy image of nanoparticle lysosomal escape, ×100.

**Figure 7:**
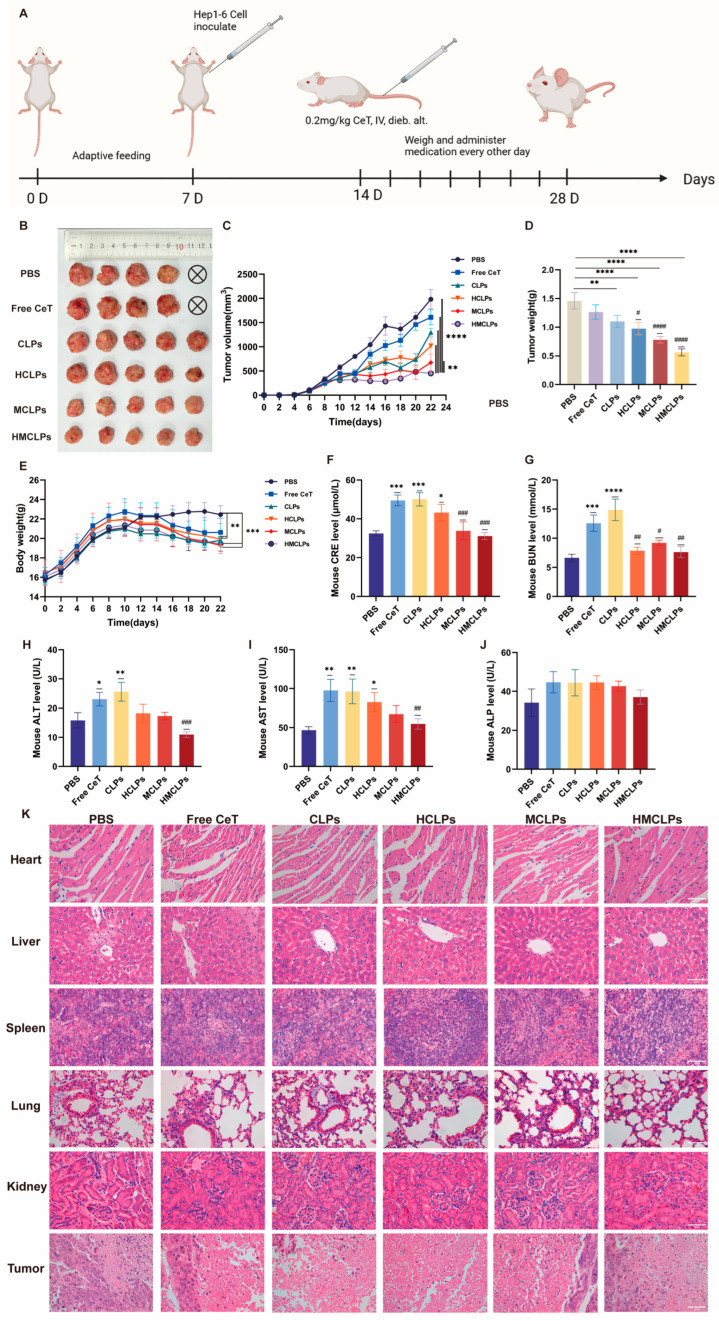
In vivo antitumor activity of tumor-bearing Balb/c-nu mice treated with various nanoparticles. (**A**) Flow chart of in vivo antitumor technology; (**B**) photos of tumor size; (**C**) tumor volume; (**D**) tumor weight; (**E**) body weight changes; (**F**) results of CRE levels; (**G**) results of BUN levels; (**H**) results of ALT levels; (**I**) results of AST levels; (**J**) results of ALP levels; (**K**) H&E staining results of various organs, ×400. Mean ± SD; *n* = 5; * *p* < 0.05, ** *p* < 0.01, *** *p* < 0.001, and **** *p* < 0.0001 compared to PBS group; ^#^ *p* < 0.05, ^##^ *p* < 0.01, ^###^ *p* < 0.001, and ^####^ *p* < 0.0001 compared to free CeT group.

**Figure S5:**
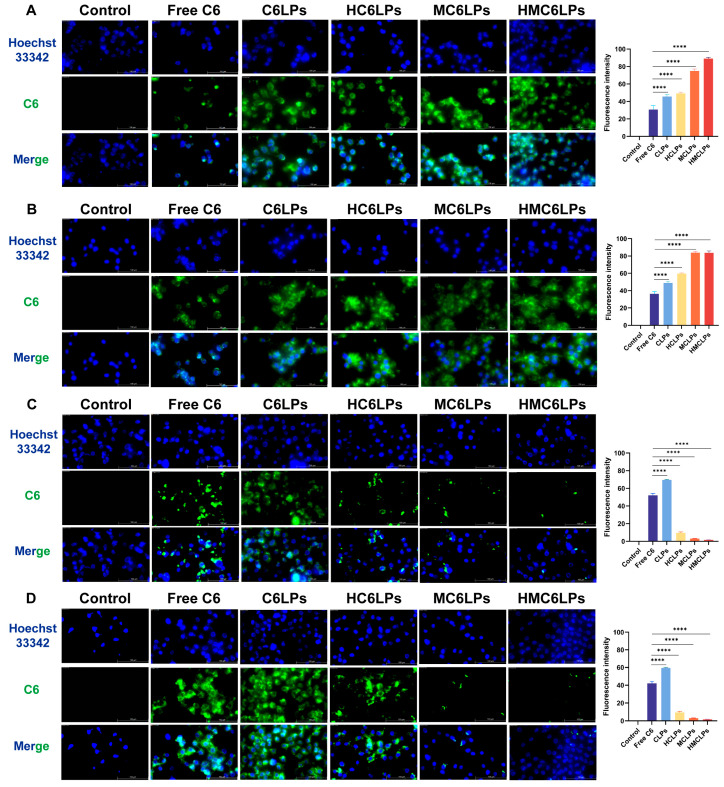
(**A**) The uptake of nanoparticles in each group in tumor cells Hep1-6, the result of incubation for one hour; (**B**) The uptake of nanoparticles in each group in tumor cells Hep1-6, the result of incubation for three hours; (**C**) The uptake of nanoparticles in each group in normal liver cells AML-12, the result of incubation for one hour; (**D**) The uptake of nanoparticles in each group in normal liver cells AML-12, the result of incubation for three hours. ×100. Mean ± SD (*n* = 3); **** *p* < 0.0001 compared to free C6 group.

The authors state that the scientific conclusions are unaffected. This correction was approved by the Academic Editor. The original publication has also been updated.
