# Supplementary material for: Celastrol-Loaded Hyaluronic Acid/Cancer Cell Membrane Lipid Nanoparticles for Targeted Hepatocellular Carcinoma Prevention
Source: Cells. 2024 Nov 4;13(21):1819. doi: 10.3390/cells13211819 (PMC11545145; doi:10.3390/cells13211819)
Supplement: Supplementary file 1 [file cells-13-01819-s001.zip › cells-3245336-supplementary.pdf]

cells

MDPI

Supplementary Materials

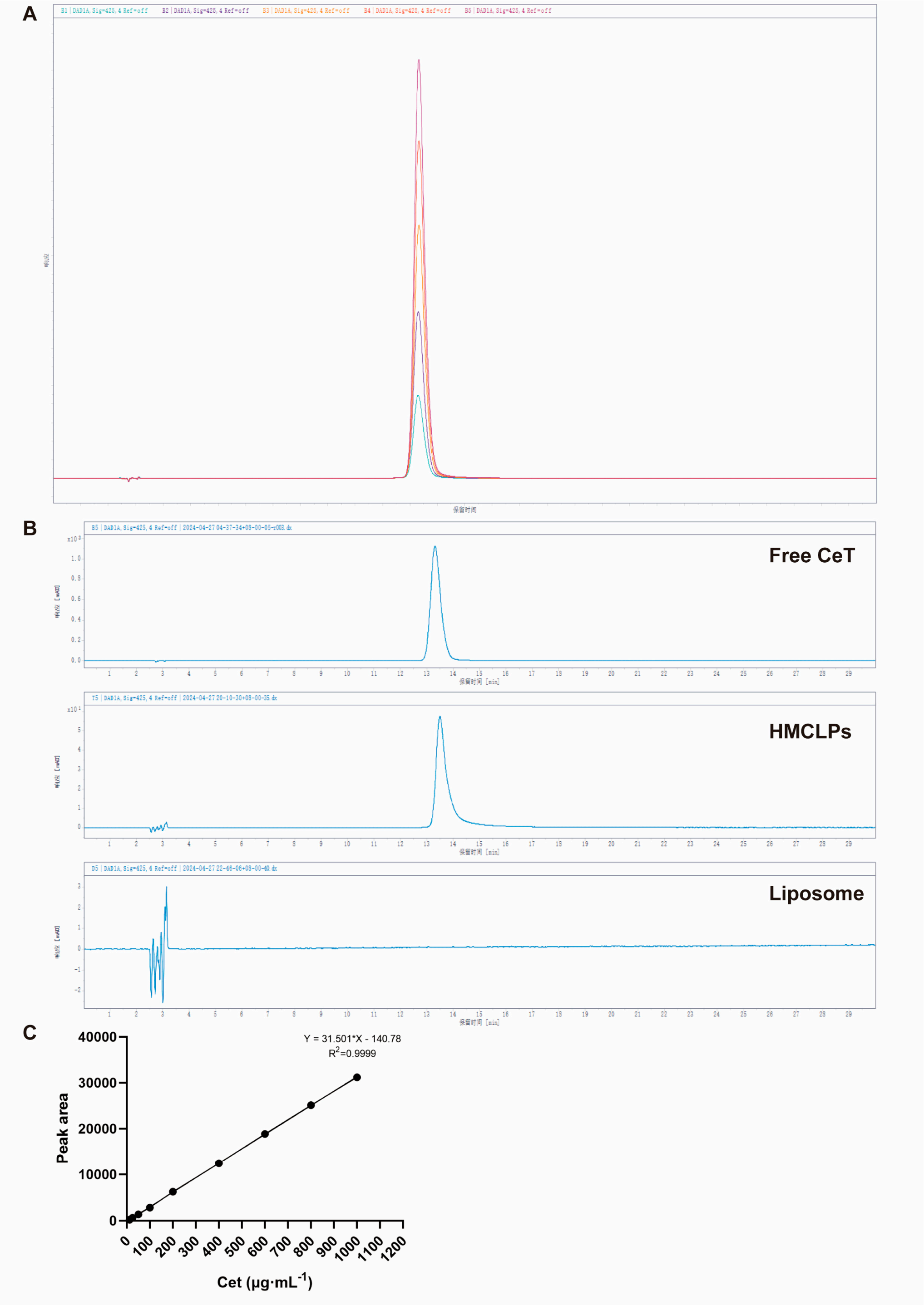

Figure S1. A HPLC detection results of celastrol at different concentrations; B Results of free celastrol, HMCLPs and liposome HPCL; C HPLC detection standard curve of celastrol.

Table S1. Investigation on the precision of HPLC detection method for celastrol.

Precision experiment results

Number of measurements	1	2	3	4	5
Peak area	552.993	1111.964	2375.468	5111.709	9758.647
	544.449	1109.649	2416.495	5101.525	9764.295
	561.427	1117.012	2394.192	5134.692	9810.238
RSD (%)	1.535%	0.338%	0.857%	0.332%	0.289%

Table S2. Stability study of HPLC detection method for celastrol.

Results of stability experiments

Number of measurements	1	2	3	4	5
Peak area	464.868	1093.096	2493.256	5011.704	9795.325
	473.195	1110.311	2501.394	5020.491	9810.002
	471.661	1102.654	2499.366	5014.268	9789.992
RSD(%)	0.943%	0.783%	0.170%	0.090%	0.106%

Table S3. Repeatability study of HPLC detection method for celastrol.

Repeatable experiment results

Measurement time(d)	1	2	3	4	5
Peak area	4776.127	4810.199	4815.294	4793.594	4792.282
	4789.659	4811.215	4792.598	4789.249	4792.697
	4790.197	4809.297	4789.649	4810.297	4801.949
RSD(%)	0.167%	0.020%	0.292%	0.232%	0.114%
			0.235%		

Table S4. Investigation on sample recovery rate of HPLC detection method for celastrol.

Sample recovery results

Amount added(μg)	Measured quantity(μg)	Recycling rate(%)	RSD(%)
	20.126	100.63	
20	19.972	99.86	0.595%
	19.892	99.46	
	39.149	97.87	
40	39.954	99.88	1.056%
	39.749	99.37	
	78.957	98.69	
80	79.431	99.28	0.539%
	79.812	99.76	
	159.459	99.66	
160	159.983	99.98	0.303%
	160.427	100.26	
	319.458	99.83	
320	319.692	99.90	0.166%
	318.678	99.58	

Table S5. Particle size, zeta potential and encapsulation efficiency of different samples

The comparison of particle size, zeta potential, and encapsulation efficiency of different samples

	Size(nm)	Zeta potential(mV)	PDI	EE(%)
LPs	80.61±2.14	33.6±1.2	0.209±0.021	0±0
CLPs	84.47±3.14	37.2±0.8	0.248±0.009	81.24±2.31
HCLPs	110.03±4.41	17.6±2.4	0.238±0.012	84.01±1.94
MCLPs	109.3±4.94	-28.6±2.1	0.232±0.004	87.69±1.62
HMCLPs	128.5±5.84	-16.4±0.9	0.233±0.011	92.16±2.08

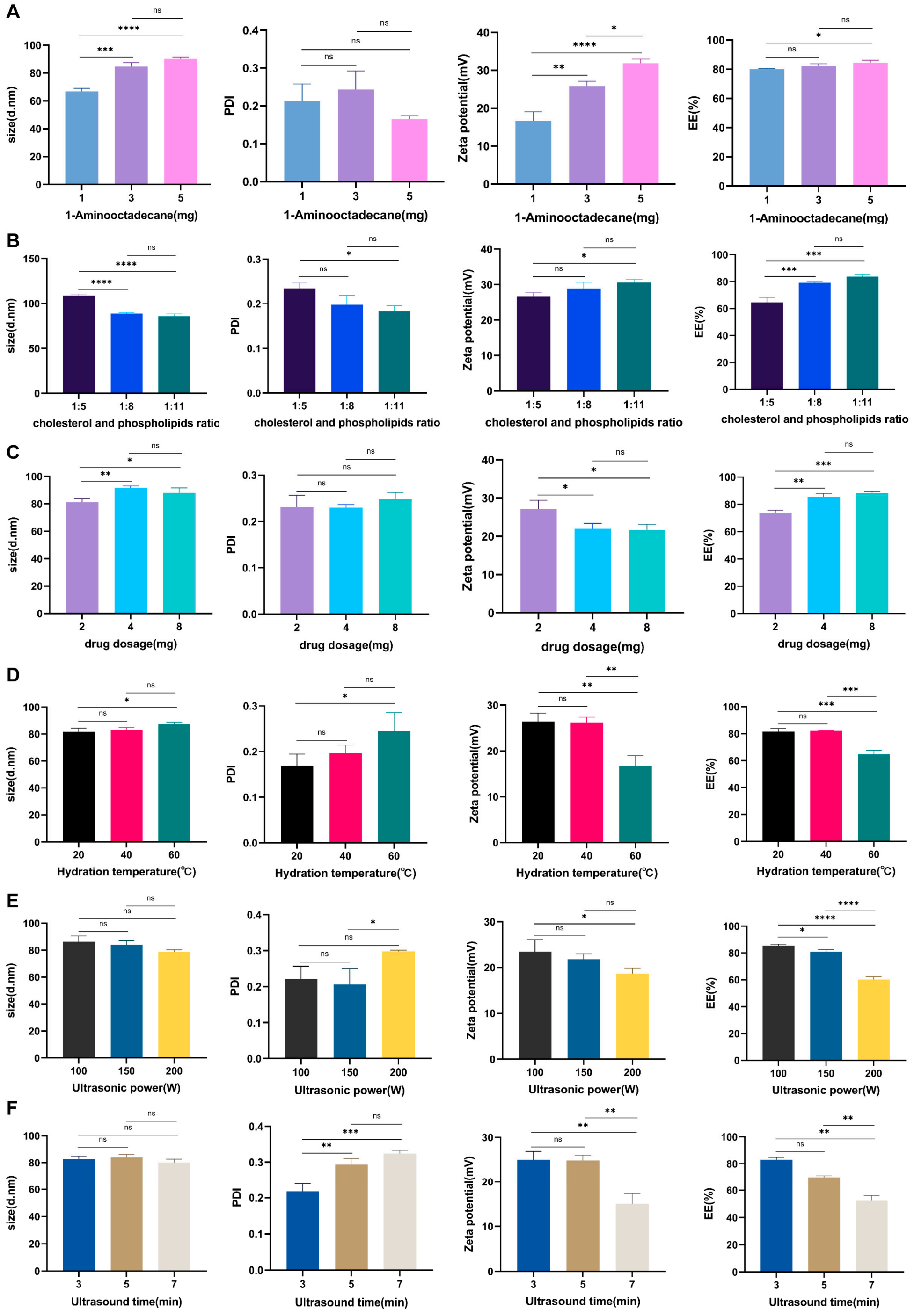

Figure S2. A Effect of 1-aminooctadecylamine addition on nanoparticle size, PDI, potential, and encapsulation efficiency; B Effect of cholesterol to lecithin ratio on nanoparticle size, PDI, potential, and encapsulation efficiency; C Effect of dosage on nanoparticle size, PDI, potential, and encapsulation efficiency; D Effect of hydration temperature on nanoparticle size, PDI, potential, and encapsulation efficiency; E Effect of ultrasound power on nanoparticle size, PDI, potential, and encapsulation efficiency; F Effect of ultrasound time on nanoparticle size, PDI, potential, and encapsulation efficiency. Mean ± SD (n=3); Statistical significance is denoted as follows: The *ns* ($p > 0.05$), *$p < 0.05$, **$p < 0.01$, ***$p < 0.001$, ****$p < 0.0001$.

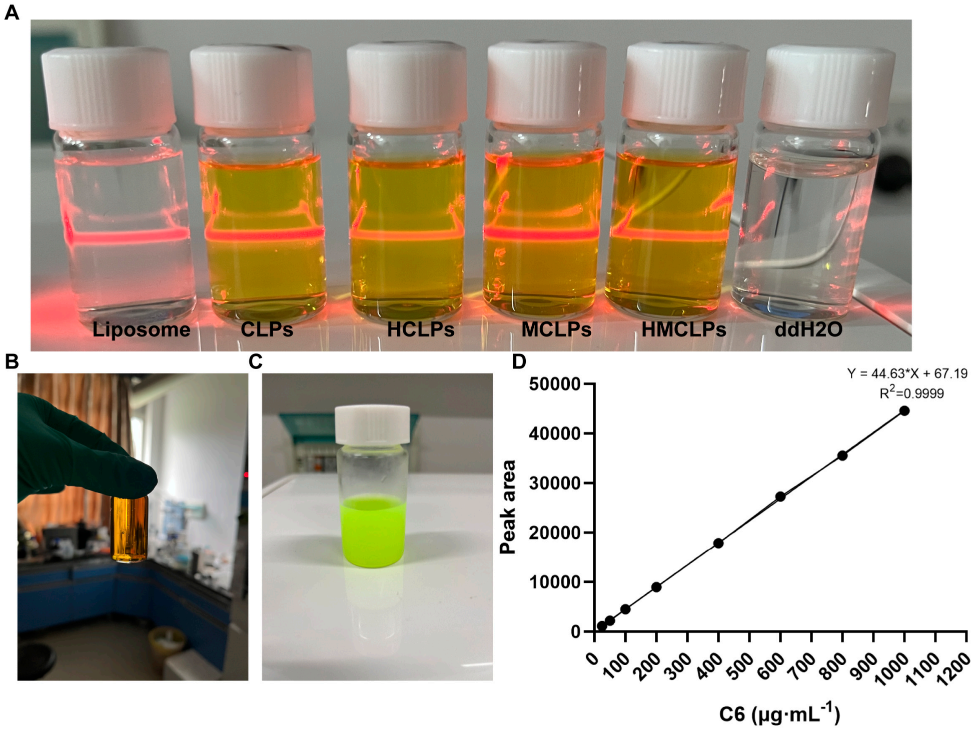

Figure S3. A The results of the Tyndall effect of nanoparticles in each group; B The appearance of HMCLPs; C The appearance of HMC6LPs; D The HPLC detection standard curve of C6.

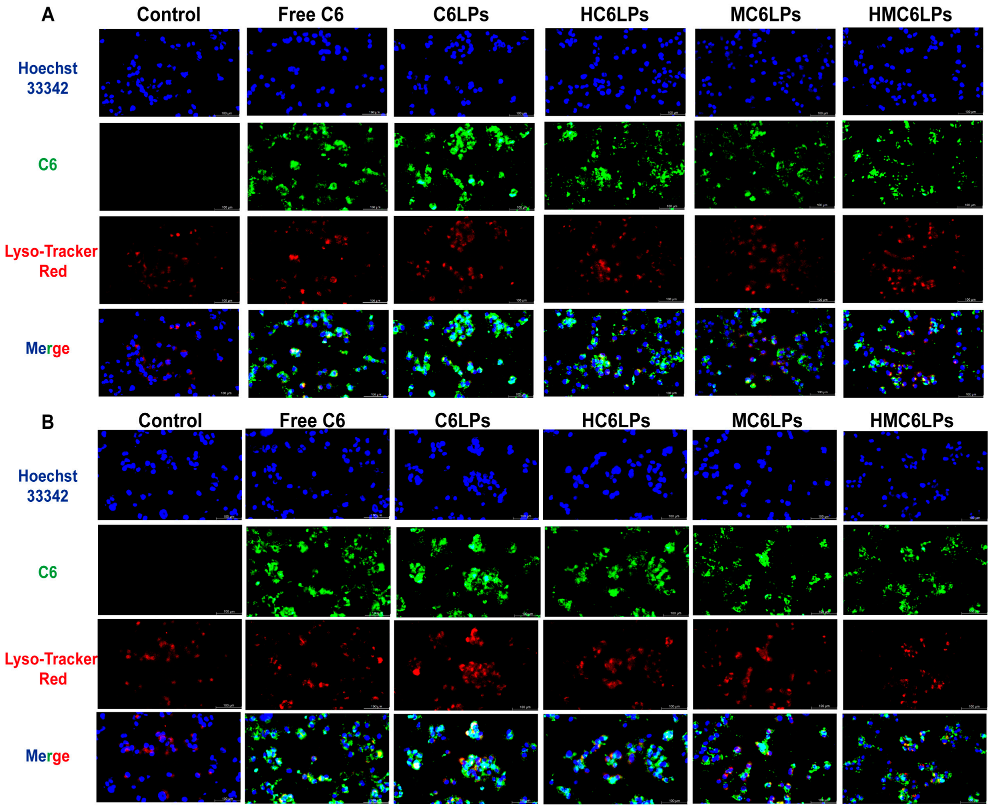

Figure S4. A: The escaping lysosomal uptake of nanoparticles in each group in tumor cells Hep1-6, the result of incubation for one hour; B: The escaping lysosomal uptake of nanoparticles in each group in tumor cells Hep1-6, the result of incubation for three hours. ×100.

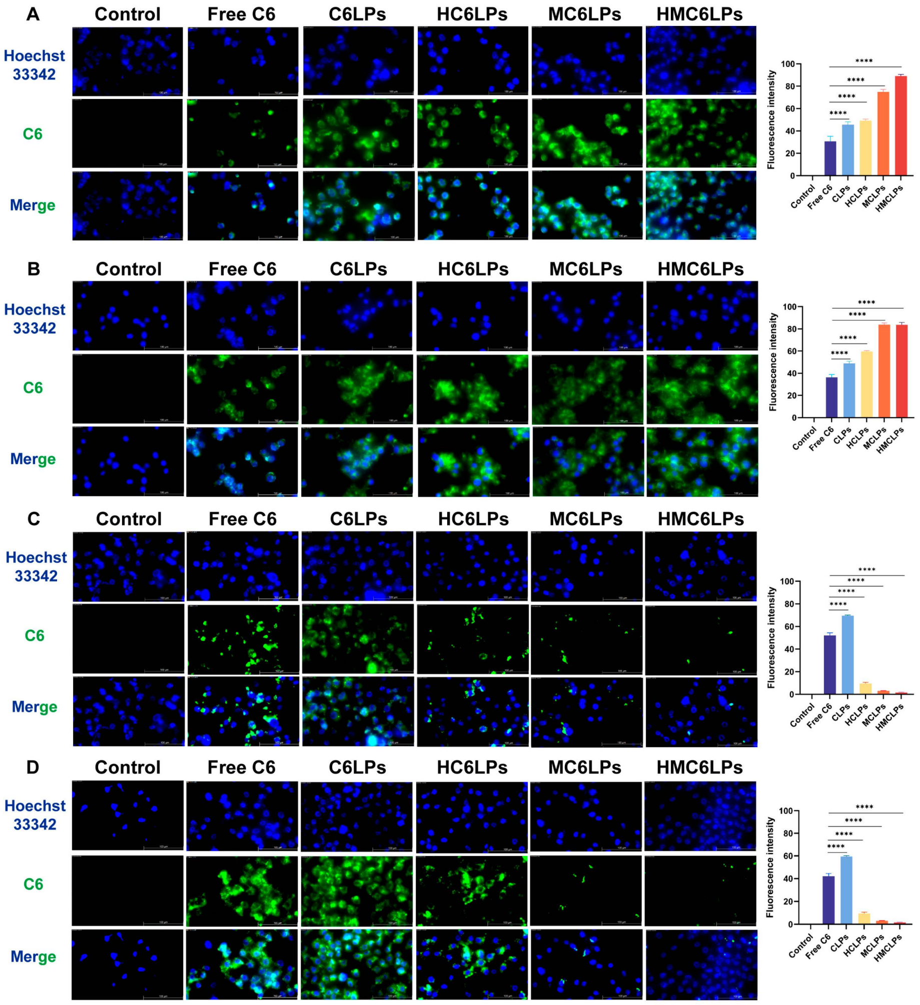

Figure S5. A The uptake of nanoparticles in each group in tumor cells Hep1-6, the result of incubation for one hour; B The uptake of nanoparticles in each group in tumor cells Hep1-6, the result of incubation for three hours; C The uptake of nanoparticles in each group in normal liver cells AML-12, the result of incubation for one hour; D The uptake of nanoparticles in each group in normal liver cells AML-12, the result of incubation for three hours. ×100. Mean ± SD (n=3); ****P<0.0001 compared to free C6 group.

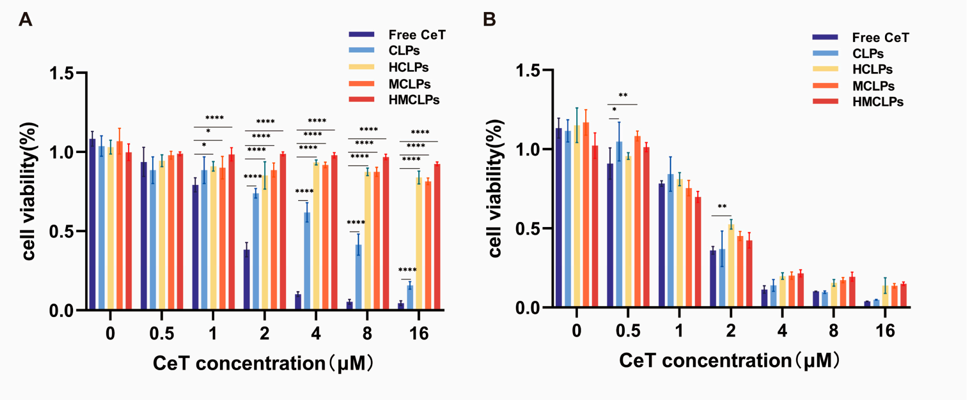

Figure S6. A CCK8 results of AML-12 cells at 48h; B CCK8 results of Hep1-6 cells at 48h. Mean ± SD (n=3); *p < 0.05, **p < 0.01, ***p < 0.001, ****p < 0.0001

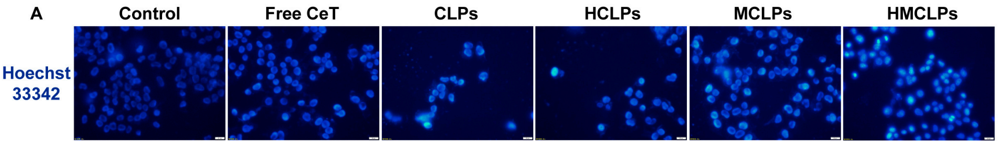

Figure S7. Hoechst 33342 staining results of cells after treatment with different nanoparticles. ×100.

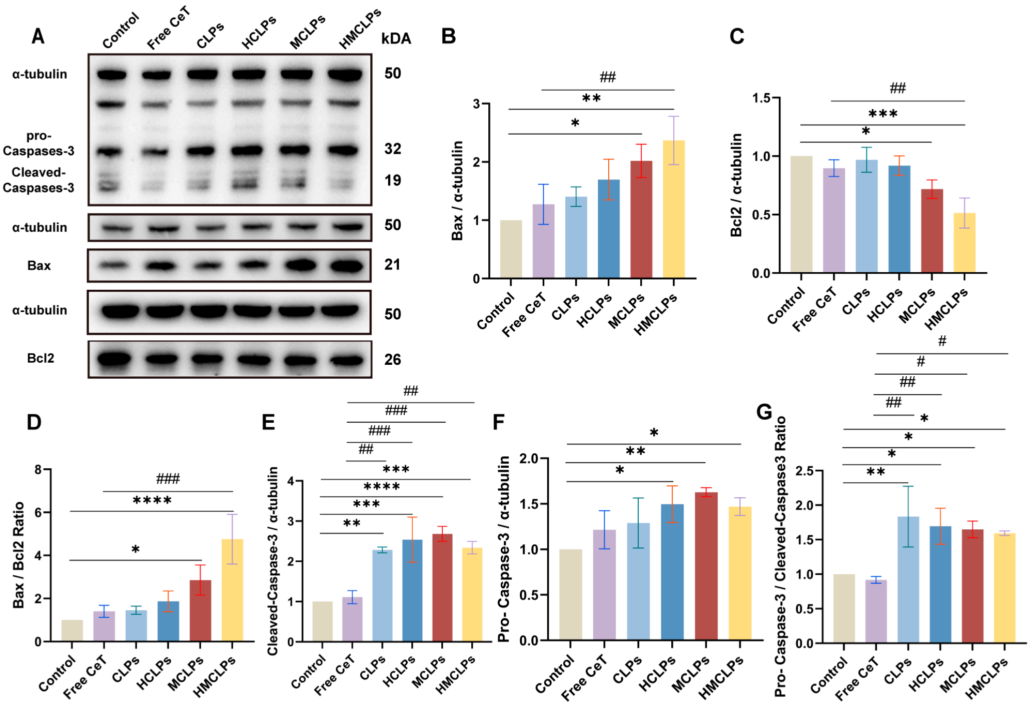

Figure S8. Western blot analysis of apoptotic markers in different treatment groups. (A) Representative immunoblots of pro-caspase-3, cleaved caspase-3, Bax, Bcl-2, and α-tubulin as a loading control. (B) Quantification of Bax expression normalized to α-tubulin. (C) Quantification of Bcl-2 expression normalized to α-tubulin. (D) Bax/Bcl-2 ratio, representing the pro-apoptotic/anti-apoptotic balance, demonstrates a significant increase in MCLPs, and HMLCPs groups, indicating enhanced apoptotic potential. (E) Quantification of cleaved caspase-3 normalized to α-tubulin. (F) Pro-caspase-3 expression normalized to α-tubulin. (G) The pro-caspase-3 to cleaved caspase-3 ratio suggests relative caspase activation across groups. Mean ± SD (n=3); Statistical significance is denoted as follows: *$p < 0.05$, **$p < 0.01$, ***$p < 0.001$, ****$p < 0.0001$ compared to the control; #$p < 0.05$, ##$p < 0.01$, ###$p < 0.001$, ####$p < 0.0001$ between Free CeT groups.
